# Evaluation of the analgesic efficacy of ultrasound-guided quadratus lumborum block for laparoscopic renal surgery: a randomized controlled trial

**DOI:** 10.1186/s12871-025-03268-8

**Published:** 2025-08-09

**Authors:** Lei Shen, Xiangjun Liu, Yuxiang Meng, Chenyang Shi, Zhibiao Xu, Yu Wang, Kang Zhou, Su Liu, Linlin Zhao

**Affiliations:** 1 Department of Anesthesiology, The Affiliated Hospital of Xuzhou Medical University, NO. 99 Huaihai Road, Quanshan District, Xuzhou City, 221002 China; 2 Jiangsu Province Key Laboratory of Anesthesiology, Xuzhou Medical University, Xuzhou, China; 3 Department of Anesthesiology, The Third Affiliated Hospital of Soochow University, Changzhou, China

**Keywords:** Analgesic efficacy, Ultrasound-guided quadratus lumborum block, Laparoscopic renal surgery

## Abstract

**Purpose:**

To evaluate the analgesic efficacy of ultrasound-guided quadratus lumborum block (QLB) for laparoscopic renal surgery.

**Methods:**

Sixty-eight patients who underwent unilateral laparoscopic renal surgery were enrolled. All patients were separated into two groups: group QLB (*n* = 34) and group C (*n* = 34). Patients in the QLB group underwent unilateral anterior QLB before the induction of anesthesia. Patients in group C underwent laparoscopic renal surgery with no block. Postoperative NRS scores at rest and during movement at 6, 12, 24, 48 h were assessed. Remedial analgesia and the incidence of adverse reactions within 48 h after surgery were recorded. The quality of recovery was compared at 24 and 48 h after the operation via the postoperative quality recovery scale.

**Results:**

At 6, 12, 24 h postoperatively, the QLB group had lower resting and moving NRS scores than did the C group (*P* < 0.05). The QLB group had lower numbers of PCAs, remedial analgesia and incidences of nausea and vomiting within 48 h (*P* < 0.05). The time to first walk and exhaust was shorter in the QLB group (*P* < 0.05). The recovery rates of nociceptive and emotional factors at 24, 48 h in the QLB group were greater (*P* < 0.05). The recovery rates of the activities of daily living (ADL) factors at 24 h in the QLB group were greater (*P* < 0.05).

**Conclusions:**

QLBs are more effective at alleviating pain in patients undergoing laparoscopic renal surgery, which is beneficial for reducing the use of opioids, alleviating adverse reactions, improving the quality of recovery.

## Introduction

Laparoscopy has gradually become the first choice for renal surgery in place of conventional open surgery. Although modern laparoscopic surgical techniques are used, the incidence of pain in patients undergoing renal surgery is still high. The causes of pain after laparoscopic renal surgery are multifaceted. Incisions in the lower abdomen, kidney injury, ureteral colic and urethral discomfort may lead to the development of postoperative pain in patients [1].

As a new technique of fascial plane block, QLB exerts an analgesic effect via the spread of local anesthetics in the thoracolumbar fascia and has been widely used in combined anesthesia and postoperative analgesia in lower abdominal surgery and lower limb surgery [2–4]. By blocking the lumbar and sympathetic nerves in the thoracic paravertebral space and thoracolumbar fascia, QLBs exert analgesic effects on both abdominal somatic and visceral pain [5]. QLB has a better and longer analgesic effect than transversus abdominis plane block (TAPB). Thus, this kind of block applies particularly for intraperitoneal and retroperitoneal surgery, especially laparoscopic renal surgery [6].

Consequently, this study aimed to evaluate the analgesic efficacy of ultrasound-guided QLB for laparoscopic renal surgery, investigate its impact on the quality of recovery and explore new protocols for intraoperative and postoperative analgesia.

## Methods

### Study design

The study was designed as a prospective, assessor-blinded randomised controlled trial. This study was approved by the Ethics Committee of the Medical Ethics Committee of the Affiliated Hospital of Xuzhou Medical University (approval number: XYFY2023-KL150-02) and was registered at chictr.org.cn (ChiCTR2300074778).All subjects were provided with written informed consent to participate in this study and all experiments were performed in accordance with relevant guidelines and regulations.

### Participants

A total of 68 patients scheduled to undergo selective unilateral laparoscopic renal surgery at the Affiliated Hospital of Xuzhou Medical University were included. The inclusion criteria were as follows: age between 25 and 65 years; American Society of Anesthesiologists physical status of 1 or 2; body mass index (BMI) of 18.5 ~ 30.0 kg/m^2^; and no central nervous system diseases before surgery; and uncomplicated airway anatomy suitable for laryngeal mask airway (LMA) use (as determined by preoperative airway assessment). The exclusion criteria included severe coagulation disorders; American Society of Anesthesiologists physical status ≥ 3; local infection at the puncture site; allergies to analgesics or local anesthetics; serious complications, such as cardiac, liver, kidney, cerebral diseases or psychiatric disorders; history of chronic pain; peripheral neurological disorders or paresthesia in dermatomes of the innervation zone; and inability to cooperate with the numerical rating scale (NRS) and postoperative quality recovery scale (PQRS) measurements; history of gastroesophageal reflux; and anticipated surgery duration > 120 min (to ensure airway stability with LMA).

### Randomization and clinding

Patients were randomized into two groups using a computer-generated simple randomization chart by an independent researcher. The investigator opened the sequentially numbered opaque envelop for each participant to determine the group allotment: the ultrasound-guided QLB group (QLB group) and the control group (C group). Participants and outcome assessors were blinded to group allocation, while anaesthesia providers could not be blinded because of the signifcant diferences between the anaesthetic techniques.

### Anesthesia procedure

All patients were given mask oxygen (5 L/min) by convention. Intravenous access was established, and electrocardiography (ECG), heart rate (HR), non-invasive blood pressure, peripheral oxygen saturation (SpO_2_), and the bispectral index (BIS) were monitored in all patients. Radial artery puncture was conducted for arterial blood pressure monitoring. All patients were not given any premedication before anesthesia. The sealed group assignment envelope was opened in the preparation room by a nurse who was not associated with the rest of the trial.

#### Quadratus lumborum block

**Fig. 1:**
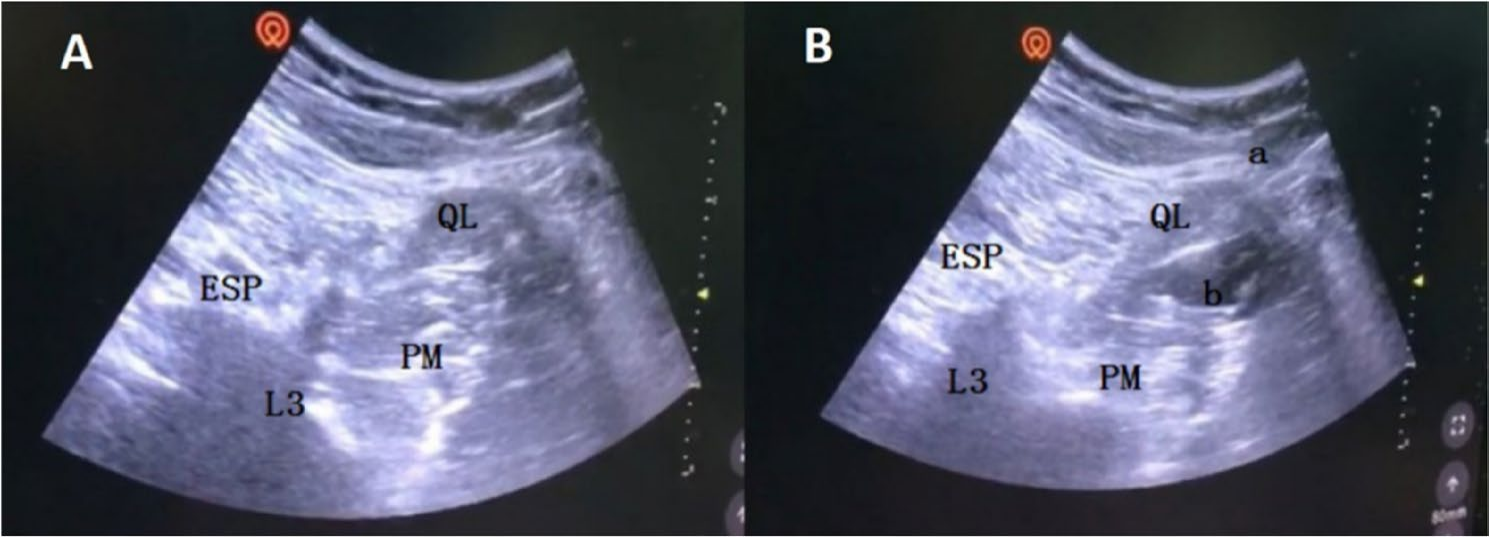
USG QLB. **A** The quadratus lumborum (QL) muscle is what the transverse process points to. **B** The local anesthetic was injected between the psoas major (PM) and quadratus lumborum (QL) muscles and spread in the thoracolumbar fascial interspace. ESP, erector spinae; QL, quadratus lumborum; PM, psoas major; L3, transverse process of the third lumbar vertebra; a, needle trajectory; b, local anesthetics

Ultrasound-guided quadratus lumborum block (USG QLB) was performed half an hour before the induction of anesthesia in patients in the QLB group. Surgical method: Patients were placed in the lateral position with the operative side facing upward. The anterior and lateral abdominal walls and the back region for nerve block anesthesia on the operative side were sterilely prepared and covered with a disposable surgical hole towel. The ultrasound equipment DOCKING CART was used for probe location. The low-frequency convex array probe was placed on the midaxillary line at the L2 level between the 12th rib and the anterior superior iliac spine. With the transverse processes visible via ultrasound, the quadratus lumborum can be found connected with the tip of the transverse process, and the surrounding structures (psoas major muscle, erector spinae muscles and kidney) can be identified. Local infiltration anesthesia was administered with lidocaine. Using an oblique anterior approach, the needle was inserted posterior to the ultrasound transducer. Under ultrasound guidance, it was advanced through the quadratus lumborum muscle and positioned within the fascial plane between the quadratus lumborum and psoas major muscles using the anterior QLB approach. The total dose of ropivacaine was 0.375%, and 30 ml was subsequently injected after a negative aspiration test for blood in the thoracolumbar fascial interspace. The spread of local anesthetic in the thoracolumbar fascia could be visualized via ultrasound (Fig. 1). The efficacy of the block was assessed by an evaluator blinded to the group assignment at 20 min post-injection, and success was determined by a comparative sensory block of the skin dermatomes. A satisfactory nerve block could be achieved when the sensory block plane covered T10 ~ L1 (the anterior and lateral abdominal wall between the 12th rib and the inguinal canal), and obvious hypoesthesia occurred. All ultrasound-guided QLB procedures in the QLB group were performed by an associate chief anesthesiologist. Patients in group C received no block.

All patients received standardized general anesthesia as follows: induction with etomidate 0.3 mg/kg, midazolam 0.05 mg/kg, sufentanil 0.5 µg/kg, rocuronium 0.9 mg/kg; Laryngeal mask airway (LMA) insertion was performed. Pneumoperitoneum pressure was maintained at ≤ 13 mmHg to minimize the risk of LMA leakage.Controlled mechanical ventilation via LMA with the following ventilator parameter settings: tidal volume (VT) 6 ~ 8 ml/kg; respiratory rate (RR) 12 ~ 15 breaths/min; oxygen 100% 2 L/min; maintenance of anesthesia with inhalation of 1%~2% sevoflurane; continuous infusion of propofol 4 ~ 6 mg/kg/h; remifentanil 0.3 ~ 0.5 µg/kg/min; cis-atracurium 0.1 mg/kg/h; target-controlled infusion concentration of maintenance drugs adjusted according to the surgical requirements; and patient response, BIS 40 ~ 60; and P_ET_CO_2_ 35 ~ 45 mmHg (1 mmHg = 0.133 kPa). The infusion of cisatracurium was discontinued when the wound was closed. Baseline blood pressure and heart rate were recorded when blood pressure and heart rate measurements were obtained preoperatively. We used a ± 20% fluctuation range from baseline to maintain stable blood pressure and heart rate via the intraoperative use of phenylephrine, urapidil and atropine. The LMA was removed once the patient was fully awake, with tidal volume and minute ventilation returning to baseline levels.When the patient’s modifed Aldrete score was ≥ 9, they could be discharged from the PACU.

Postoperative analgesia: PCIA: sufentanil 100 µg + dolasetron 25 mg. The total volume in the device was 100 ml, with no background infusion, 2 ml of PCA each time and a 15-min lockout interval. Bucinnazine hydrochloride (100 mg) was administered intramuscularly as remedial analgesia if the NRS score was still greater than 4 after patient-controlled analgesia. Patients received the pain assessment 10 min after the remedial measures were taken until the NRS score was not greater than 3.

### Outcome measurements

This study evaluated postoperative pain intensity at rest and during movement using the Numerical Rating Scale (NRS) at 6, 12, 24, and 48 h after surgery (primary outcomes), along with secondary outcomes including patient-controlled analgesia (PCA) demands and patients requiring rescue analgesia within 48 h, postoperative adverse reactions (nausea/vomiting, arrhythmia, local anesthetic toxic reactions [tinnitus, metallic taste], respiratory depression, pruritus, hip flexion/knee extension weakness, sustained hypotension), time to first ambulation and flatus, length of hospital stay, and postoperative recovery quality assessed via the Postoperative Quality Recovery Scale (PQRS).The PQRS is a measurement tool used to evaluate postoperative recovery quality in multiple domains and time periods [7]. It is a brief test that is feasible for tracking recovery from immediate to long-term time periods in patients of varying ages, languages, cultures, and physical conditions. It mainly consists of five domains (physiological factors, nociceptive factors, emotional factors, activities of daily living factors and cognitive factors). Postoperative values are compared with baseline values tested 1 day preoperatively to obtain dynamic observations of postoperative recovery quality. The Chinese version of the PQRS [8] was used in this study. Assessments were performed at 24 and 48 h postoperatively across these domains.Observational variables included baseline/preoperative demographics (age, sex, BMI, hypertension/diabetes status), surgery type, and surgical duration.

### Sample size calculation

According to previous data [9], the VAS score at rest 24 h after laparoscopic renal surgery is 3.7 ± 2.0. The difference was considered to be clinically significant when the VAS score of the experimental group was two points lower than that of the control group. A 90% power was provided. The required sample size was 23, as calculated by PASS 15.0 software (Stata Corp. LP, College Station, Texas, USA). A dropout rate of 30% is allowed considering the possible dropout data, and the required sample size is theoretically 33. In this study, 34 patients were included in each group, and the total sample size was 68.

### Statistical analysis

Statistical analysis was performed via SPSS 23.0 software (IBM SPSS, Chicago, Illinois, USA). The quantitative data with a normal distribution are expressed as the means ± SDs ($\overline x\pm s$). Between-group comparisons were tested with a t test when homogeneity of variance existed and a t test when heterogeneity of variance was present. Repeated measures analysis of variance was used for within-group comparisons. The quantitative data that did not fit a normal distribution are presented as medians (interquartile ranges) [M(Q1, Q3)], and between-group comparisons were performed via the Mann‒Whitney U test. The qualitative data are expressed as frequencies (n) or percentages (%) and were analyzed with the χ2 test. *P* < 0.05 was considered significant.

## Results

**Fig. 2:**
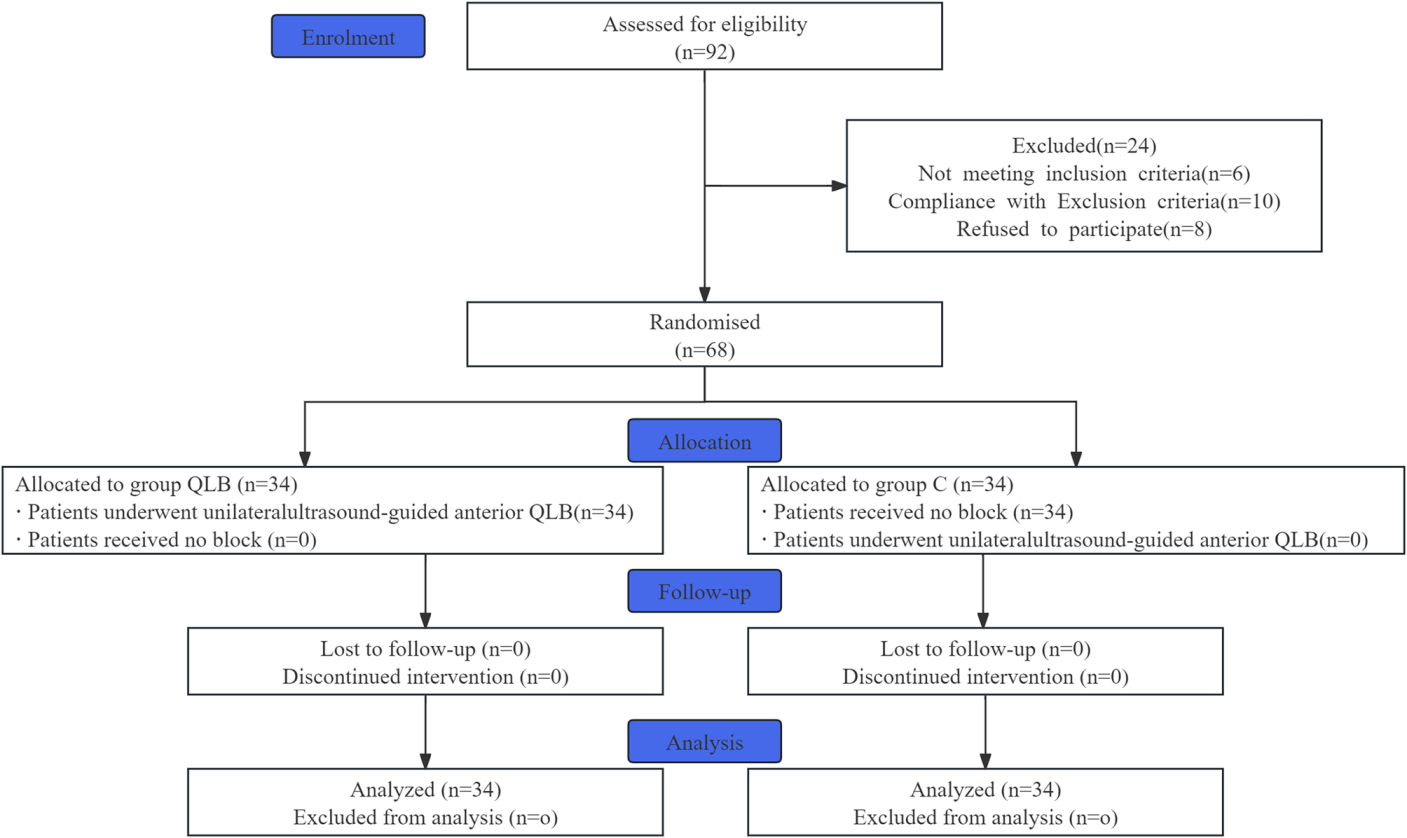
Flowchart

Overall, 92 patients were assessed for eligibility, and 68 patients were included and randomized (Fig. 2).

1. Comparisons of general information: There were no significant differences in general information (age, sex, BMI, hypertension, diabetes, duration or type of surgery) between the two groups (*P* > 0.05). Intraoperative intravenous maintenance drug consumption (propofol and remifentanil) markedly decreased in the QLB group (*P* < 0.05, Table 1).

**Table 1:** Comparisons of general information of two groups

Items	Group QLB(*n* = 34)	Group C(*n* = 34)	*P* value
Sex (male/female)	17/17	16/18	0.808
Age(yr)	50.88 ± 9.28	48.41 ± 11.30	0.310
BMI(kg/m^2^)	24.99 ± 3.27	24.73 ± 3.25	0.830
Hypertension	14(41)	16(47)	0.625
Diabetes	9(27)	10(29)	0.787
Duration of surgery (min)	96.15 ± 31.07	100.32 ± 30.78	0.580
Propofol (mg)	280(210 ~ 450)^*^	550(400 ~ 700)	< 0.0001
Remifentanil (ug)	500(250 ~ 1000)^*^	1500(825 ~ 3000)	< 0.0001
Type of surgery			
Decompression surgery of renal cyst unroofing	11(32)	11(32)	1.000
Partial ephrectomy	9(26)	11(32)	0.595
Partial renalectomy	7(21)	5(15)	0.525
Radical ephrectomy	7(21)	7(21)	1.000

All values shown are mean ± SD or median (IQR) or n (%) as appropriate

*SD* standard deviation, *IQR* inter-quartile range, BMI body mass index

^*^P < 0.05 compared with group C
2. Comparisons of postoperative analgesic efficacy: At 6, 12 and 24 h postoperatively, the QLB group had significantly lower resting and moving NRS scores than did the C group (*P* < 0.05). The resting and moving NRS scores were statistically similar (*P*>0.05, Table 2) at 48 h in the two groups.

**Table 2:** Comparisons of postoperative NRS of two groups at different time points

Group	*n*	Resting NRS (scores)				Moving NRS (scores)			
		6 h	12 h	24 h	48 h	6 h	12 h	24 h	48 h
Group QLB	34	3(2 ~ 4)^*^	2(1.75 ~ 4)^*^	1(1 ~ 2)^*^	1(0 ~ 2)	3(3 ~ 4)^*^	3(2.75 ~ 4)^*^	2(2 ~ 3)^*^	1.5(0 ~ 2)
Group C	34	4(3 ~ 6)	4(3 ~ 4.25)	3(2 ~ 3.25)	2(0 ~ 2.25)	5(4 ~ 6)	5(4 ~ 6)	4(3 ~ 4)	2(0 ~ 2.25)

All values shown are median (IQR)

*NRS* numeric rating scale

^*^P < 0.05 compared with group C
3. Comparisons of postoperative adverse reactions and recovery between the two groups revealed that no incidence of arrhythmia, local anesthetic toxic reactions (tinnitus, metallic taste), respiratory depression, pruritus, weakness of hip flexion or knee extension, or sustained hypotension occurred. The time to first walk (22.57 ± 2.40 h) and first exhaust (26.15 ± 3.54 h) in the QLB group was significantly lower than the time to first walk (25.28 ± 2.09 h) and first exhaust (29.62 ± 2.40 h) in the C group (*P* < 0.05). The length of hospital stay after surgery was statistically similar between the two groups (*P* > 0.05). Compared with Group C, Group QLB had a lower number of PCAs and patients who received remedial analgesia within 48 h (*P* < 0.05). The incidence of nausea and vomiting in Group QLB (8.8%) was significantly lower than that in Group C (32.4%). (*P* < 0.05, Table 3).

**Table 3:** Comparisons of postoperative recovery of two groups

Group	Time to first walk (h)	Time to first exhaust (h)	length of hospital stay after surgery(h)	Number of PCA	Nausea and vomiting
Group QLB	22.57 ± 2.40^*^	26.15 ± 3.54^*^	73.79 ± 2.52	0(0 ~ 1.25)^*^	3(8.8)^*^
Group C	25.28 ± 2.09	29.62 ± 2.40	74.74 ± 2.82	2(0 ~ 3)	11(32.4)

All values shown are mean ± SD or median (IQR) or n (%) as appropriate

*PCA* patient controlled analgesia

^*^P < 0.05 compared with group C
4. Postoperative recovery quality was assessed via the PQRS at baseline (1 day preoperatively), 24 h, and 48 h. Preoperative PQRS baseline values were 100% (as patients were in their normal physiological state). Postoperative recovery rates were calculated as the percentage of patients returning to baseline function in each domain.

Intragroup comparisons showed QLB group’s nociceptive factor recovery rates decreased to 23.53% at 24 h but improved to 67.65% at 48 h, vs. control group’s 2.94% and 32.35%, respectively. QLB group also had faster emotional factor (61.76% at 24 h vs. 35.29%) and ADL factor (76.47% at 24 h vs. 47.06%) recovery. Intergroup comparisons confirmed QLB group had higher recovery rates in nociceptive (*P* < 0.05), emotional (*P* < 0.05), and ADL factors (*P* < 0.05) at both time points. Physiological and cognitive factors showed no significant intergroup differences (Table 4).

**Table 4:** Comparisons of recovery quality of respective factors at 24 and 48 h postoperatively of two groups

PQRS	Group QLB		Group C	
	24 h	48 h	24 h	48 h
Physiological factors	85.29	94.12	73.53^#^	91.18
Nociceptive factors	23.53^*#^	67.65^*#^	2.94^#^	32.35^#^
Emotional factors	61.76^*#^	94.12^*^	35.29^#^	67.65^#^
ADL factors	76.47^*#^	97.06	47.06^#^	100.00
Cognitive factors	97.06	100.00	94.12	100.00

All values shown are n (%)

*PQRS* postoperative quality recovery scale, *ADL* activities of daily living

^#^*P* < 0.05 compared with baseline values

^*^P < 0.05 compared with group C

## Discussion

Patients after laparoscopic renal surgery suffer moderate to severe pain, which has negative impacts on their mood, daily activities and early functional recovery. Thus, effective postoperative analgesia is the key to early postoperative recovery in patients [10].

There are four different approaches for ultrasound-guided quadratus lumborum block based on the location of injection [11]: QLB 1 (injection lateral to the QL muscle) [12], QLB 2 (injection posterior to the QL muscle) [13], QLB 3 (injection anterior to the QL muscle, transmuscular QLB) [14] and QLB 4 (injection inside the fascia of the QL muscle) [15]. Notably, different approaches to QLB lead to different analgesic efficacies. Li et al. [16] reported that the lateral and posterior approaches of QLB for laparoscopic renal surgery improved postoperative analgesia. Currently, laparoscopic renal surgery is mostly performed via a posterior laparoscopic approach. The incisions are located on one side of the lower anterior and the lateral abdominal wall. As described by Tamura et al. [17], intramuscular QLB induced no sensory block but had an analgesic effect on the lower midabdomen. The anterior QLB approach allows local anesthetics to spread into the thoracic paravertebral space and thoracolumbar fascia to block the ventral rami of the spinal nerves and the ilioinguinal and iliohypogastric nerves, covering the afferent innervation of the incision (anterior rami of the spinal nerves from T10 to L1), reaching adequate analgesic efficacy for moderate to severe postoperative pain [18, 19]. Renal and ureteral pain are conducted through the visceral sensory nerves of T10 ~ L1 and T10 ~ L2, respectively, the nerve fibers of which run along the corresponding segment of the sympathetic nerve [20, 21]. QLB can achieve effective analgesia for intraoperative and postoperative pain by blocking the corresponding segment of the sympathetic nerve in the thoracic paravertebral space and thoracolumbar fascia [22]. Therefore, USG QLB has common analgesic effects on both abdominal somatic and visceral pain to obtain intraoperative analgesia. Moreover, Rao Kadam et al. [23, 24]. extended the application of the QLB block by performing the ultrasound-guided transmuscular quadratus lumborum block catheter technique in abdominal surgery and achieved sensory dermatomal coverage from T4 to L1. Good anesthetic conditions can be conducive to surgical procedures when combined with general anesthesia via a laryngeal mask. Effective postoperative analgesia can also reduce the negative impact of pain on sleep, early walking and early gastrointestinal functional recovery.

The primary outcomes demonstrated that QLB significantly reduced postoperative pain scores at rest and during movement up to 24 h, supporting its role as an effective analgesic modality. Secondary outcomes further revealed reduced opioid consumption, faster functional recovery, and improved quality of recovery, aligning with enhanced recovery after surgery (ERAS) principles.

Intraoperative intravenous maintenance drug consumption (propofol and remifentanil) was markedly lower in the QLB group than in the C group. The NRS score at rest and movement in the QLB group were lower than those in the C group at 6, 12, and 24 h postoperatively. The QLB group had a significantly lower number of PCAs and patients who received remedial analgesia within 48 h than did the C group did. All of the above results suggested that the QLB could provide satisfactory intraoperative and postoperative analgesia, greatly reducing the consumption of intraoperative and postoperative analgesic drugs. This finding was similar to the results of Corso et al. [25]. The results from Hansen et al.’s [26] study revealed no opioid consumption reduction in total laparoscopic hysterectomy. The negative results suggested that the QLB is not suitable for all lower abdominal surgeries. The incidence of postoperative adverse responses, such as nausea and vomiting and the time to first walk and exhaust, was significantly lower than that in group C, which therefore suggested that the adverse responses and negative impacts on recovery caused by analgesic drugs in group QLB were also reduced. Compared with Group C, Group QLB had lower resting and moving NRS scores at 48 h postsurgery, but the NRS scores were statistically similar between the two groups. The lower NRS pain score might be related to the alleviation of the postoperative inflammatory response and greater pain relief.

The recovery rates of nociceptive factors, emotional factors and ADL factors at 24 h and nociceptive factors and emotional factors at 48 h in the QLB group were greater than those in the C group. This difference was possibly caused by the alleviation of postoperative pain, nausea and vomiting by the QLB. Therefore, feelings of anxiety and depression were relieved. The improvement in postoperative comfort also contributed to daily activities such as dressing and walking. The recovery rates of physiological factors and cognitive factors in the two groups were statistically similar at 48 h postsurgery. Considering the relationship with the type of surgery, further study is needed to confirm this.

Effective analgesia can remarkably reduce the intraoperative and postoperative consumption of intravenous analgesic drugs such as opioids and NSAIDs. Moreover, disturbances in gastrointestinal function and the risk of respiratory depression, asphyxia and hypoxia can also be decreased. Prolonged effective analgesia can promote early activities and improve patient comfort, satisfaction and quality of recovery. The individual multimodal analgesia provided for patients coincides with ERAS (enhanced recovery after surgery) strategies of perioperative management on the basis of evidence-based medicine [27]. USG QLB combined with general anesthesia provides effective intraoperative and postoperative analgesia for patients and lessens the harm induced by the perioperative stress response. As an important part of ERAS, pain treatment is conducive to early postoperative rehabilitation.

Despite the promising results, this study has several limitations. First, the relatively small sample size (34 patients per group) may limit the generalizability of the findings, and larger multicenter trials are needed to validate these outcomes. Second, the follow-up period was limited to 48 h postoperatively, leaving the long-term analgesic effects and recovery quality unexplored. Third, although PQRS were used to evaluate the quality of recovery, other potential confounding factors, such as preoperative psychological state and medication use, were not systematically evaluated. Future studies, including extended follow-up periods, broader demographic inclusion, and comprehensive biomarker analyses, can further clarify the role of QLB in enhancing the recovery pathway.

## Conclusion

The results of this randomized controlled trial show that ultrasound-guided anterior QLB is more effective in alleviating the pain of patients undergoing laparoscopic renal surgery, contributing to reducing the use of opioids intraoperatively and postoperatively, alleviating adverse reactions and improving the quality of recovery. QLB, which is consistent with ERAS strategies, is a good choice for patients undergoing laparoscopic renal surgery.

## Data Availability

Research data can be available from the corresponding authors on reasonable request.

## Abbreviations

QLB: Quadratus lumborum block
BMI: Body mass index
HR: Heart rate
IBP: Invasive arterial blood pressure
SpO2: Peripheral oxygen saturation
BIS: Bispectral index
VT: Tidal volume
RR: Respiratory rate
PETCO2: Partial pressure of carbon dioxide
PCIA: Patient-controlled intravenous analgesia
NRS: Numeric rating scale
PQRS: Postoperative quality recovery scale
ERAS: Enhanced recovery after surgery
TAPB: Transversus abdominis plane block
NSAIDs: Non-steroidal anti-inflammatory drugs
ADL: Activities of daily living
ECG: Electrocardiography
